# COVID-19 Impact on the Doctor-Patient Relationship: Patient Perspectives on Emergency Physician Empathy and Communication

**DOI:** 10.30476/BEAT.2021.89058.1216

**Published:** 2021-07

**Authors:** Sophia Aguirre, Kristen M. Jogerst, Zachary Ginsberg, Sandeep Voleti, Puneet Bhullar, Joshua Spegman, Taylor Viggiano, Jessica Monas, Douglas Rappaport

**Affiliations:** 1 Mayo Clinic, Alix School of Medicine, Phoenix, AZ, USA; 2 Department of General Surgery, Mayo Clinic Arizona, Phoenix, AZ, USA; 3 Department of Emergency Medicine, Mayo Clinic Arizona, Phoenix, AZ, USA

**Keywords:** COVID-19, Empathy, Communication, Doctor-patient relationship

## Abstract

**Objective:**

To investigate in how the current COVID-19 pandemic affects patient’s perceptions of emergency physician empathy and communication.

**Methods:**

Patients cared for by Emergency Department physicians with the lowest satisfaction scores were surveyed within one week of discharge via phone. Using questions from the Consultation and Relational Empathy (CARE) survey, patients rated their satisfaction with their Emergency provider’s empathy and communication on a scale of 1 to 5 and provided feedback on how the patient-provider interaction could be improved. Demographic data and patient responses to CARE survey questions were compared between pre-COVID-19 and during COVID-19 time. Patient’s open-ended responses were analyzed for themes related to the impact of COVID-19 on the patient-provider relationship.

**Results:**

Patient median quantitative scores were 5 (4-5) across all five questions of pre-COVID-19 and 5 (4-5) during COVID-19 for all questions except two (showing care and compassion), median 5(5-5). Female patients rated provider empathy and communication lower than mens. There was no differences across age strata. A shift in provider focuses to COVID-19 only care (N=3), and an understanding of the stress on healthcare processes (N=13) from open-ended responses themes emerged of patients who want to minimize interactions within the emergency department (N=3).

**Conclusions:**

The external factor of the current pandemic did not negatively impact patient’s satisfaction scores. Many patients express leniency and gratitude for emergency providers during this challenging time. Their responses seem to mirror current societal views of frontline healthcare workers.

## Introduction

A greater emphasis has been placed on patient satisfaction in healthcare with the implementation of the Hospital Consumer Assessment of Healthcare Providers and Systems (HCAHPS) survey, which measures patient satisfaction in hospitals nationwide. Patient satisfaction has been associated with improved patient adherence to treatment and better clinical outcomes [1]. In the emergency department, prior literature has shown that patients frequently cite components of the doctor-patient relationship, such as communication and empathy, as factors that influence their satisfaction with the healthcare experience [2, 3]. Further literature emphasizes the importance of provider care and compassion, as physicians who self-report higher empathy levels often have higher patient satisfaction scores. In addition, effective physician communication improves patient adherence to medical guidelines [4, 5]. 

While this prior work has placed the emphasis on improving provider interpersonal skills to improve patient satisfaction, it is well-understood that the doctor-patient relationship is multifactorial with many external factors contributing to how both the provider and patient view a given medical encounter [6]. Physician’s perceptions of their patients’ attitudes also impact this relationship, and providers display more patient-centered care to patients who they believe are better communicators [7]. Patient demographics and physician bias towards a given patient can also influence satisfaction, given patient perceptions of any relationship, including with healthcare team members, are constructed based on prior experiences and their mental model [8]. Variables ranging from patients’ sex to physician attire can influence patient-reported satisfaction with their care [9-11]. A number of institution-related factors outside of the patient and physicians’ control can also impact their relationship. Increased numbers of nursing staff and nurse to patient ratios correlate with improved patient satisfaction [8,11], while the perception of a busy and loud environment is associated with decreased patient satisfaction [9, 12-14].

In recent months, medicine has experienced an unprecedented external factor with the outbreak of the coronavirus pandemic. In the early stages of the COVID-19 pandemic, the healthcare community and general public anxiously awaited the development of a vaccine as well as evidence-based interventions that improve outcomes in patients with COVID-19. However, at present as vaccine rollout remains in progress, the emphasis still remains on containing outbreaks via social distancing, mask use, symptom detection, isolation, and contact tracing, and clinically supportive treatment for patients infected with the novel coronavirus [15]. Given paucity of curative interventions, this time of uncertainty imposes stress on both providers and patients. While the pandemic has imposed many negative stressors on the American healthcare system, it has also influenced how society views healthcare workers, with many cities referring to healthcare workers as heroes and celebrating their hard work through media publications and nightly ovations [16, 17]. 

Given that the patient-provider relationship is impacted by external factors and the landscape of our local healthcare system is rapidly evolving in response to COVID-19, our study aims to investigate what impact this pandemic has on patient satisfaction. We compare perceptions of emergency medicine physicians’ empathy and communication before COVID-19 and during COVID-19. In addition, it aims to examine how patients’ perceptions of physician empathy and communication potentially are impacted by multiple contextual factors, such as baseline patient demographic data versus healthcare system changes implemented in response to the pandemic. 

## Materials and Methods

Institutional Review Board (IRB) approval was sent to Mayo Clinic Arizona’s IRB and exemption status was obtained given the minimal risk to subjects and quality improvement nature of the project. Using the Consultation and Relational Empathy (CARE) survey, a previously validated tool to measure patient’s perceptions of empathy and the research team developed a five-question survey inquiring about patients’ perception of their recent physician-patient interaction in our emergency department [18]. The five questions were chosen specifically to highlight aspects of patient expectations in an emergency department to visit as laid out in the Villancourt, *et al*., [19]. Questions included “How good was the physician at: 1. really listening, 2. showing care and compassion, 3. fully understanding your concerns, 4. explaining things clearly, and 5. making a plan of action with you.” Research team members pilot-tested the survey and updated it based on content clarity feedback and phone survey flow, prior to beginning patient enrollment. 

We purposefully sampled patients cared by the four emergency department providers with the lowest patient satisfaction scores to determine if these providers were also receiving low empathy scores from patients and to target a potential empathy and communication quality improvement intervention for the lower performing physicians. A report of patients discharged by these selected providers was de-identified and distributed to the research team each week. of The research team members trained in phone survey methodology and qualitative interviewing techniques who read the survey questions to each patient. At the start of each phone call, patients’ permission for participating was obtained verbally. Therefore, patients who agreed to participate were asked to rate their agreement with each of the five CARE survey statements on a five-point scale from 1 (“not at all”) to 5 (“completely agree”). (See Supplement 1 for a complete copy of the survey). After the quantitative portion of the survey, patients were asked to open-ended feedback for further assess how, if at all, empathy and communication could be improved in our local emergency department. These qualitative responses were recorded, transcribed, de-identified, and organized in Dropbox until they were later pooled together for final analysis within Dedoose software. Patients’ quantitative survey answers were assigned a randomly generated research number ID to exclude any names, Medical Record Numbers, or other identifying information prior to record the responses in Google spreadsheets. Then, the pooled data was reformatted in Microsoft excel and uploaded into STATA for final analysis (StataCorp. 2019. Stata Statistical Software: Release 16. College Station, TX: StataCorp LLC). 

Establishing Pre vs. During COVID-19 Cohorts

To respond a potential increase in patient volume secondary to the COVID-19 pandemic, our hospital erected a tent on 21.03.2020. It was used to triage COVID-19 testing with nasopharyngeal swabs and assess the COVID-19 symptoms that required discharge or transfer to isolated care rooms in the main emergency department. Also, to decrease the patient and provider traffic within the emergency department and hospital and to limit an exposures in patient and health care worker. The COVID-19 tent opened on 21.03.2020 in front of the emergency department main entrance and conspicuously symbolized the hospital’s response to the COVID-19 pandemic. It was easily appreciated by all incoming emergency patients and the local community because of its visibility. For this reason, we selected 21.03.2020 as the cutoff for our pre-COVID19 vs. post-COVID19 analyses. 

Statistical Analysis

We compared patients’ pre-COVID19 vs. during-COVID19 demographic data using descriptive statistics. Age was compared between the two groups using an unpaired t-test. Patient’s sex was compared using Chi-Square. Given ordinal-scale survey data, such as Likert scales can be analyzed under the assumptions of normal and non-normal data spread, patients’ quantitative responses to the CARE survey items were summarized using both means (standard deviations) and medians (interquartile ranges). Responses between the two groups were compared with One way analysis of variance (ANOVA), the Kruskal-Wallis test, and a Wilcoxon-Rank Sum test. All three statistical methods resulted in almost identical p values, also, for simplicity only p values for the Wilcoxon Rank Sum test were reported below. Age was organized into four strata: 0-25, 26-50, 51-75, and >75 years to compare survey responses across different demographic groups and scores were compared using the Kruskal-Wallis test. Men and women patients’ responses to each of the five CARE survey questions was compared using a Mann-Whitney U test. A *p*-value of 0.05 was used as the threshold for statistical significance. 

Qualitative Analysis

Free responses to the open-ended question at the end of the phone survey were filtered by SA to isolate responses directly related to the COVID-19 pandemic or external, non-provider specific factors. Once separated, these pandemic-related responses were inductively coded and collapsed into overarching themes. The thematic analysis of all free responses was completed via multiple deliberations between SA and KJ. Comparisons of responses across themes were rooted in the grounded theory of how external stressors and societal perceptions of healthcare workers can potentially affect the emergency department physician-patient relationship during the pandemic. 

## Results

Demographic Data

Of the 708 patients purposefully sampled, 221 agreed to participate in the phone survey: 78 in the pre-COVID-19 time period and 143 following the implementation of the COVID-19 triage tent. Patient participants in the pre-COVID-19 versus during COVID-19 times were not different based on average age: 56.3 years vs. 56.7 years, respectively. The two groups also did not differ based on patient’s sex which 55.1% were female in the pre-COVID-19 group vs. 48.2% during COVID-19 (Table 1). 

**Table 1:** Patient Demographics Pre versus During COVID-19

**Demographic**	**Pre-COVID19**	**During COVID19**	***p *** **value** ^a^
N (%)	78 (35.3)	143 (64.7)	
Mean Age (SD)	56.3 (19.7)	56.7 (20)	0.88
Age Range	18-94	17-94	
Female (%)	43 (55.1)	69 (48.2)	0.33
Male	35 (44.9)	74 (51.8)	

^a^ Age was compared between the two groups using an unpaired t-test, *p* value reported is two-tailed p-value using the t distribution. Patient sex is compared using Chi-Square (Chi2=0.95). A *p* value of 0.05 was used as the threshold for statistical significance

Quantitative Responses

**Fig. 1:**
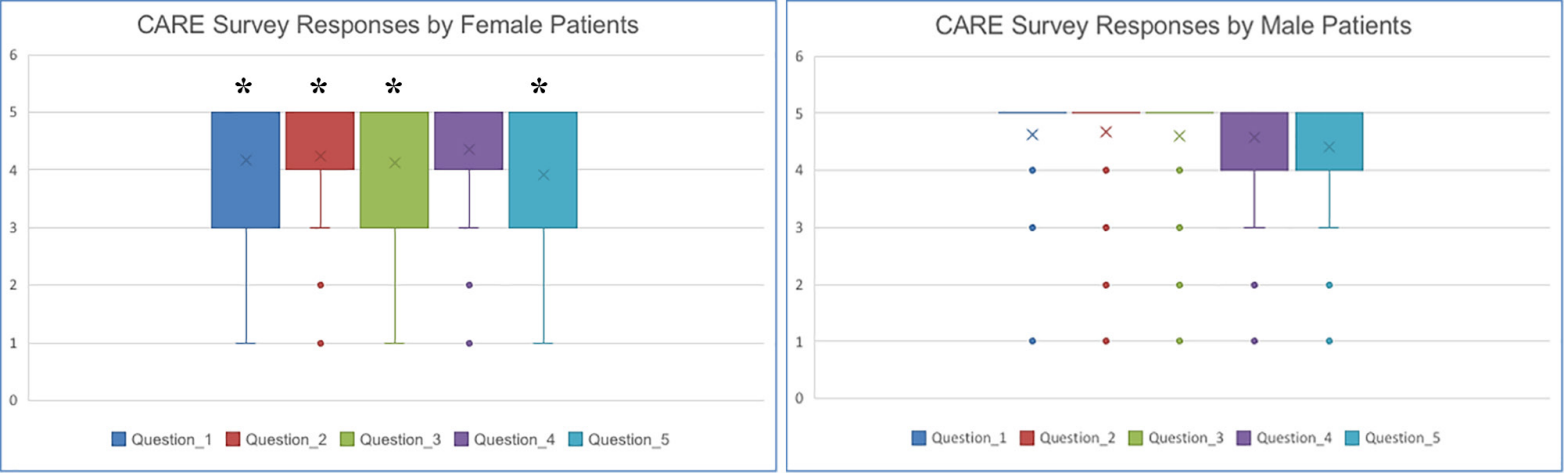
Box Plots of CARE Survey Responses by Patient Sex. *Indicated *p*<0.05 Male patients rated providers significantly higher than female patients on listening, care and compassion, understanding concerns, and a clear discharge plan (questions 1,2,3,5). Both male and female patients had a median score of 5 for all 5 survey questions. Female IQRs were 3 to 5 for listening, understanding concerns, and a clear discharge plan (questions 1,3, and 5) and fell between 4 and 5 for care and compassion and clear explanations (questions 2, 4). Male IQRs fell between 5 and 5 for listening, care and compassion, and understanding concerns (questions 1-3) and between 4 and 5 for clear explanations and a clear discharge plan (question 4,5).

**Fig. 2.**
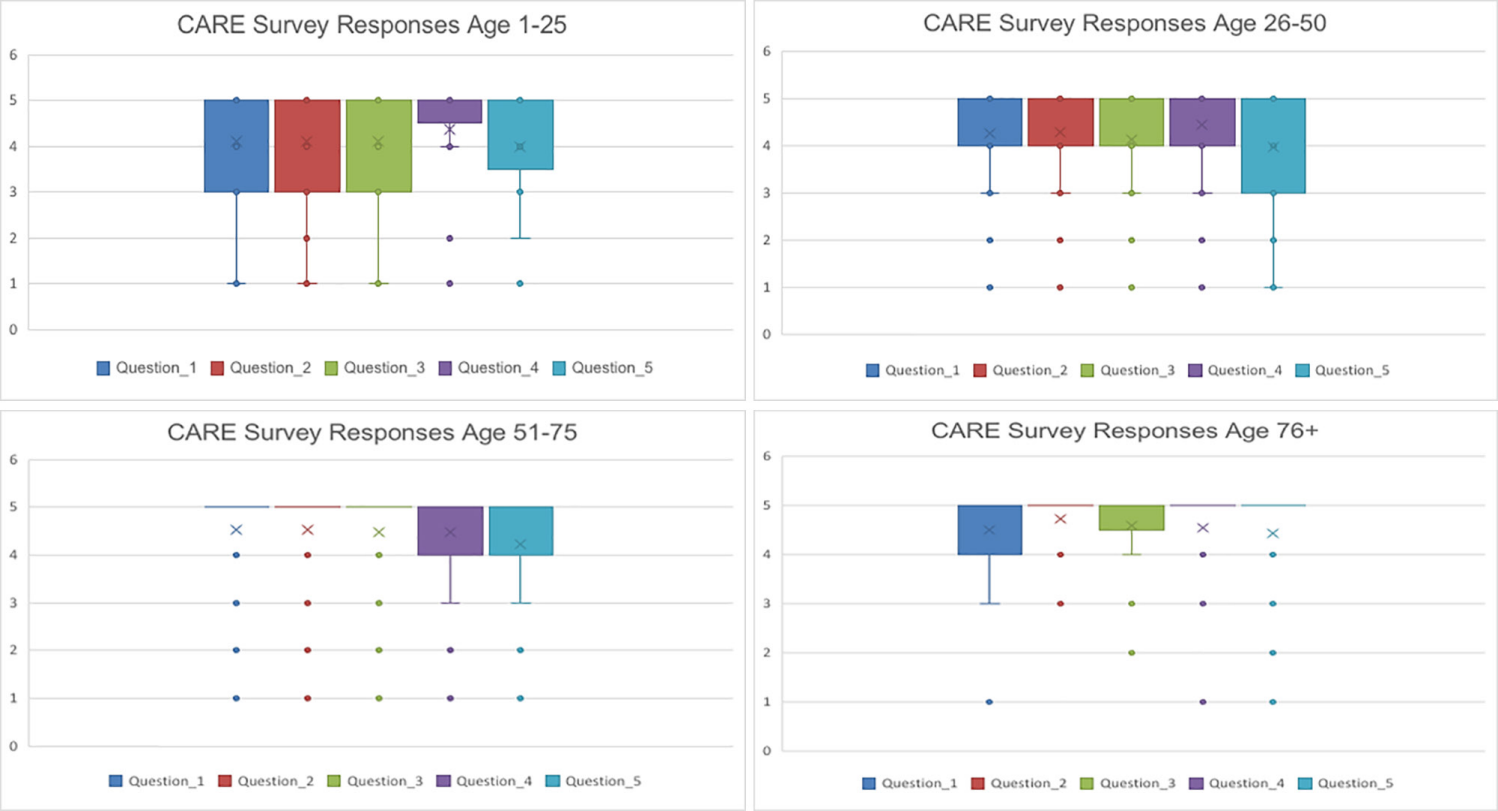
Box Plot of CARE Survey Responses by Patient Age. Survey responses were not significantly different between age groups. Patients >75 years old had the highest average survey responses for provider listening, care and compassion, understanding concerns, and a clear discharge plan (questions 1,2,3,5). The median score for all age groups was 5. Patients age 1-25 had an IQR of 3-5 for listening, care and compassion, and understanding concerns (question 1-3), an IQR of 5-5 for clear explanations (question 4), and an IQR of 5-5 for clear discharge instructions (question 5). Patients 26-50 had an IQR of 4-5 for listening, care and compassion, understanding concerns, and clear explanations (questions 1-4) and an IQR of 3-5 for a clear discharge plan (question 5). Patients 51-75 had an IQR of 5-5 for listening, care and compassion, and understanding concerns (question 1-3) and IQR of 4-5 for clear explanations and discharge plan (question 4,5). Patients >75 had an IQR of 4-5 for listening (question 1) and IQR of 5-5 for care and compassion, understanding concerns, clear explanations, and discharge plan (question 2-5).

The mean empathy and communication scores for the survey response data in the pre-COVID19 time ranged from 4.11 to 4.5. The mean empathy and communication scores for the survey response data in the post-COVID19 time ranged from 4.18 to 4.5. The median empathy and communication scores across all five questions were 5 with an interquartile range of 4-5 in the pre-COVID19 time period (Table 2). Patients’ responses did differ based on demographic data of sex, but not age. Female patients overall gave lower ratings for perceived provider empathy and communication with a median total score for the five questions of 24 (17-25) vs. their male counterparts whose median total score was 25 (23-25) (Figure 1). The four age strata did not give significantly different survey responses with median total scores of 23 (18-25) for patients age 1-25, 24 (18.5-25) for patients age 26-50, 25(22-25) for patients 51-75, and 25(22-25) for patients >75 years of age (Figure 2). 

**Table 2:** CARE Survey Responses Pre-COVID19 vs. During-COVID19

**CARE Survey Item**	**Pre-COVID19 mean (SD)**	**During-COVID19 mean (SD)**	**Pre-COVID19 median (IQR)**	**During-COVID19 median (IQR)**	***p*** ** value** ^a^
Question 1	4.23 (1.26)	4.49 (1.03)	5 (4-5)	5 (5-5)	0.110
Question 2	4.37 (1.14)	4.5 (1.06)	5 (4-5)	5 (5-5)	0.38
Question 3	4.25 (1.25)	4.42 (1.11)	5 (4-5)	5 (4-5)	0.32
Question 4	4.5 (1.03)	4.46 (1.04)	5 (4-5)	5 (4-5)	0.62
Question 5	4.11 (1.46)	4.18 (1.35)	5 (4-5)	5 (4-5)	0.91
Total	21.47 (5.43)	22.05 (5.05)	24.5 (20-25)	25 (21-25)	0.51

^a^ *p* value reported is for Mann Whitney U test comparing the pre vs. post COVID-19 groups. One-way ANOVA, Kruskal Wallis, and Mann Whitney U analyses were done to compare the two groups and given the *p* values were similar across analyses; only one *p* value is reported for simplicity

Qualitative Analysis

A total of 221 patients offered feedback to the open-ended question at the end of the survey. From this feedback, a total of 18 patients commented on COVID19-related themes and how the pandemic has affected the doctor-patient relationship. The remaining patients discussed provider-specific actions or non-pandemic specific care logistics, such as wait times, which fall outside the scope of this research project. From these free responses, three key themes emerged (Table 3).

**Table 3:** COVID-19 Impact on the Doctor-Patient Relationship

**Theme**	**Representative Quote(s)**
Minimizing Interactions	“With the Coronavirus outbreak [the] less often they come into the room, the better I guess.”
	“I also feel like I had to interact with ten different people during the time I was getting stitches, which I felt like was completely unnecessary especially considering the COVID times. If we didn’t have to switch rooms in between I could have minimized contact with other people”
	“The Emergency Department was a mess. I know Coronavirus is going around, I understand that, but the other thing was that it was disheartening that we were only allowed one visitor in the ER room.”
A Focus Only on COVID-19	“No, perfect. They did a COVID test and were very proactive.” “My big issue was that I did not have COVID and so they kicked me out of the hospital, but then I came back to the hospital the next day with an even higher fever. I feel like if the doctors had not been so focused on COVID they would not have let me go only to come back a day later and be diagnosed with viral meningitis.”
	“I was also offered a chance to take a COVID test which I thought was a good idea as well.”
Under the Circumstances	“I thought with everything going on, my visit went much better than I anticipated. Everyone was really good to me. The doctor was great, especially under the circumstances right now.”
	“There was only one thing and it wasn’t [the provider’s] fault. It’s a little different now with the virus”
	“Under the circumstances, [the provider] was okay.”
	“With this COVID 19, they are all learning on the fly. I don’t think there is anything they could have done differently to be honest with you...we all have been through a lot. The medical profession is trying to help us.”
	“I know they are busy and COVID is happening”
	“Oh, no I think they moved along pretty quickly for an ER considering the current restrictions. So I was actually quite pleased.”
	“This visit was at the beginning of the COVID virus and I worked in the ED and am familiar with how they operate. I do not understand how they could have kept their calm in the situation. The waiting room was full and the staff maintained professionalism.”
	“No, it’s during the COVID crisis so under those circumstances there was nothing else they could have done”
	“[The provider] had a lot going on and trying to be very quick and [the provider] stepped up to that but a little on the harsh side. In a couple years I went back with this and I got the same treatment I’d be surprised but we don’t know anything about Coronavirus so [the provider] just told me to self quarantine and rest up and that’s about it. But I get it, we don’t know much about this right now.”
	“All of this chaos was going on, but I think they handled the chaos very well... It’s very busy and considering that, it’s a good outcome. Under the circumstances, I don’t think [the provider] could have because it was a stressful environment. There were people literally lined up in the hallways.”
	“Everyone was so friendly…It is a hard time for everybody.”
	“I think it was mostly being unnerved that they were going through the COVID-19 stuff right now. I think [the provider] did the best [the provider] could given the circumstances.”

Three patients discussed the contact restrictions that were enforced within healthcare facilities to decrease viral transmission at the onset of COVID-19. Some highlighted of how minimizing contact and keeping social distance was important in the emergency department, while others expressed frustration with social distancing policies. One patient discussed how it was “disheartening” that she was only allowed one visitor in the room and was stressed by being distanced from her usual support network. 

Three patients commented on the perception of a heightened focus on COVID-19 during their visit, even if that was not the main concern causing them to present to the emergency department. One patient in particular reported frustrations with how the perceived focus on COVID-19 prevented physicians from potentially diagnosing her correctly. Other patients seemed to be appreciative of this increased pandemic focus and expressed gratitude that they were given the chance to receive COVID-19 testing during their visit for a separate medical issue. 

The majority of patients (n=13) expressed an understanding of how the current pandemic brought about changes to the healthcare workflow. Many patients reported comments of increased leniency towards what otherwise might have led to poor patient satisfaction ratings for providers. This flexibility in the face of a pandemic led to patients expressing concern for the emergency physicians: “It wasn’t [the provider’s] fault. It’s a little different with the virus now,” and “[the provider] was going through a lot.” This understanding and leniency extended beyond care or compassion for the provider to diagnostic expectations with a perception that knowledge of and treatment for this virus is evolving and everyone is “learning on the fly” as “we don’t know much about this [the virus] right now.” 

## Discussion

The patient-provider relationship is influenced by external context, as much as it is influenced by the patient and physician themselves. Within the context of the COVID-19 pandemic, societal perceptions of emergency medicine providers and patients’ interaction with the healthcare system have changed. This cross-sectional survey study of patients recently discharged from the emergency department, both before and during the pandemic, shows that patients’ perceptions of provider empathy and communication are positive during both time periods. Furthermore, and of equal importance, we did not see a decline in patients’ perceptions of provider empathy and communication after the onset of the COVID-19 pandemic and despite the stresses and challenges that naturally have resulted from this. Overall, quantitative scores for emergency provider empathy and communication averaged greater than four on a five-point scale, with median scores of five across all five questions before and during the pandemic. For this reason, the study was underpowered to detect a statistically significant difference if one existed. Patients’ qualitative responses expose themes consistent within the broader societal framework during the pandemic such as social distancing (minimizing interactions in the emergency department), increased awareness of the virus’s impact (a focus on COVID-19), and patient understanding of changes to healthcare delivery brought about by the COVID-19 pandemic (under the circumstances). 

Overall patients gave high ratings on perceived provider empathy and communication both before and during the pandemic despite sampling patients cared for by providers with the lowest patient satisfaction scores. These high ratings could have been biased by participant selection, as of the 708 patients identified, only 221 gave responses. To decrease likelihood of patients reporting skewed positive feedback, members of the research team explained to each patient at the beginning of the call that their answers were anonymous, members of the research team were not involved in their care, and participation would not impact their ability to receive future care within our institution. Given small effect size and positively skewed survey results, a focus on how pandemic factors affect the patient-provider relationship was more apparent in the qualitative analysis. Thematic analysis of the open-ended responses showed that the majority of patients who referenced COVID-19 in regards to impact on satisfaction with their provider did so in a way that showed leniency towards the provider amidst the current pandemic. Patient comments centered around an understanding that providers are dealing with challenging times and even if the provider did do something that would otherwise give the patient a poor perception of the physician, the patient placed blame on the current circumstances instead of the provider. Patients at our institution seemed to mirror current societal views of health care workers as heroes in light of the current pandemic [16, 17]. Within the background of a change in the societal context, it seems patients are less likely to give poor patient satisfaction ratings to their emergency providers during this difficult time. One patient expressed this sentiment best by saying, “I don’t think there is anything they could have done differently to be honest with you...we all have been through a lot. The medical profession is trying to help us.”

Beyond the pandemic, patient perceptions of emergency physician empathy and communication varied based on patient-related factors. Similar to prior work on the patient-provider relationship, our patient population’s ratings of provider satisfaction were also impacted by patient sex [9, 10]. Overall women patients rated providers lower across perceived provider listening, care and compassion, understanding of patient concerns, and a clear discharge plan (questions 1,2,3, and 5). Interestingly, our results do not agree with prior work that suggests patient satisfaction is negatively impacted by a busy and loud environment [13, 14]. Many patients referenced the busy environment caused by COVID-19; however they did so in a way that placed the provider’s actions in this context and excused some of their otherwise negatively perceived behavior. Instead of provider satisfaction scores decreasing as the emergency department became more hectic with the onset of COVID-19, scores remained positively skewed. Prior literature shows that older patients give higher provider satisfaction scores [6, 20]. However, the differences in scores between our age strata were small, and thus we were unable to detect an age influence on emergency provider satisfaction in this analysis. 

To the best of our understanding, no prior study examines how the external stress of the COVID-19 pandemic affects patient satisfaction with their emergency department care. During these unprecedented times for the medical community, patients recognize the unique stressors forced upon healthcare workers and seem to be both supportive of providers and understanding of the demands placed on them. This global external factor has not been a detriment to the patient-doctor relationship or provider satisfaction, but rather was the underlying foundation impacting patient praise for and support of emergency medicine providers. 

Patient satisfaction scores of perceived emergency provider empathy and communication remained positive despite the onset of the COVID-19 pandemic. Qualitative analysis also showed mostly positive feelings towards providers and an understanding of the evolving landscape of healthcare. Patients at our institution seem to mirror societies’ positive feelings towards frontline healthcare workers during the pandemic as well as recognition of emergency physician’s increased and evolving focus on COVID-19 and the challenges that come with this. Our study provides insight on how COVID-19 impacted patient satisfaction. Future work could include exploring patient satisfaction in the emergency department post COVID-19 to see how pandemic media attention has impacted patients’ view of frontline workers long term as well as investigating patient satisfaction surrounding other external healthcare stressors such as natural disasters to see if the same leniency applies. 

## Limitations

This study is limited in scope as we sampled patients only evaluated in and discharged from a single-institution tertiary-care emergency department. We also chose to sample patients only seen by providers with the lowest patient satisfaction scores in order to target potential quality improvement interventions prior to the arrival of COVID-19. This may have resulted in biased results that would not be generalizable for providers with higher satisfaction scores. With the arrival of the pandemic, the scope of the research question evolved to a more general focus on how external contextual factors influence the patient-provider relationship, particularly unprecedented events such as the COVID-19 pandemic. With this purposeful sampling, we may have missed out on feedback from patients evaluated by providers with overall higher satisfaction ratings. Additionally, utilization of the CARE survey has never been validated in the Emergency Department setting. Another limitation was our study was underpowered to detect a statistically significant difference between pre COVID-19 and during COVID-19 scores as we did not predict the positively skewed ratings, even amongst the poorer performing providers, across all five CARE survey questions. Given the unexpected arrival of the pandemic, our statistical analyses were also subject to unequal numbers of patients in our pre-COVID19 and during COVID-19 groups. However, with the shift in the local context, the research question also shifted in hopes of providing emergency medicine and all healthcare providers with data on how the pandemic is impacting their patients’ perceptions of their care. Lastly, because the pre-COVID-19 CARE survey scores were already so high, it is difficult to quantify the impact of the pandemic on post-COVID19 CARE survey scores.

## Funding:

This research did not receive any specific grant from funding agencies in the public, commercial, or not-for-profit sectors.

## Conflict of Interests:

None declared.
